# A proteasomal β5 subunit of *Haemonchus contortus* with a role in the growth, development and life span

**DOI:** 10.1186/s13071-023-05676-6

**Published:** 2023-03-15

**Authors:** Li He, Hong-Run Zhang, Wen-Da Di, Fang-Fang Li, Chun-Qun Wang, Xin Yang, Xiao-Fang Liu, Min Hu

**Affiliations:** 1 State Key Laboratory of Agricultural Microbiology, Key Laboratory for the Development of Veterinary Products, Ministry of Agriculture College of Veterinary Medicine, Huazhong Agricultural University, Wuhan, 430070 Hubei Province People’s Republic of China; 2 Hubei Key Laboratory of Embryonic Stem Cell Research, School of Basic Medical Sciences, Hubei University of Medicine, Shiyan, 442000 Hubei Province People’s Republic of China; 3 College of Animal Science and Technology, Guangxi University, Nanning, 530004 Guangxi Zhuang Autonomous Region People’s Republic of China

**Keywords:** Bortezomib, *Haemonchus contortus*, Immunohistochemical, Proteasomal β5 subunit, PBS-5

## Abstract

**Background:**

The proteasome in eukaryotic cells can degrade a variety of proteins and plays an important role in regulating the cell cycle, cell survival and apoptosis. The proteasome receives much attention as a potential chemotherapeutic target for treatment of a variety of infectious parasitic diseases, but few studies of proteasomes have been done on parasitic nematodes.

**Methods:**

A proteasomal β5 subunit encoding gene (named *Hc-pbs-5*) and its inferred product (*Hc*-PBS-5) in *Haemonchus contortus* were identified and characterized in this study. Then, the transcriptional profiles and anatomical expression were studied using an integrated molecular approach. Finally, a specific proteasome inhibitor bortezomib (BTZ), together with RNA interference (RNAi), was employed to assess the function of *Hc*-PBS-5.

**Results:**

Bioinformatic analysis revealed that the coding sequence of *Hc-pbs-5* was 855 bp long and encoded 284 amino acids (aa). The predicted protein (*Hc*-PBS-5) had core conservative sequences (65–250 aa) belonging to N-terminal nucleophile (Ntn) family of hydrolases. Real-time PCR results revealed that *Hc-pbs-5* was continuously transcribed in eight developmental stages with higher levels at the infective third-stage larvae (L3s) and adult males of *H. contortus*. Immunohistochemical results revealed that *Hc*-PBS-5 was expressed in intestine, outer cuticle, muscle cells under the outer cuticle, cervical glands and seminal vesicles of male adults and also in intestine, outer cuticle, cervical glands, uterine wall, eggs and ovaries of female adults of *H. contortus*. BTZ could reduce proportions of egg hatching, and the fourth-stage larvae (L4s) developed from the exsheathed L3s (xL3s) of *H. contortus*. In addition, silencing *Hc-pbs-5* by soaking the specific double-stranded RNA (dsRNA) could decrease the transcription of *Hc-pbs-5* and result in fewer xL3s developing to L4s in vitro.

**Conclusions:**

These results indicate that proteasomal β5 subunit plays an important role in the growth, development and life span of *H. contortus*.

**Graphical Abstract:**

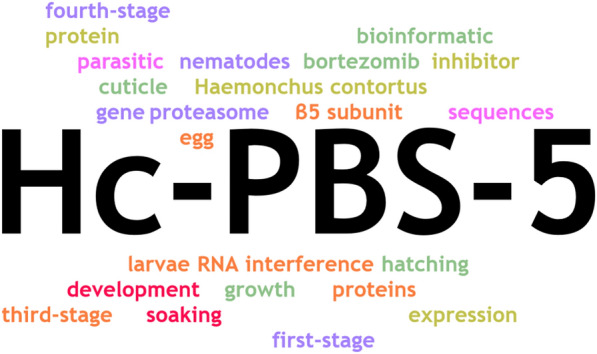

**Supplementary Information:**

The online version contains supplementary material available at 10.1186/s13071-023-05676-6.

## Background

Protein homeostasis is one of the key sites that must be preserved to keep organism balance [1, 2], which is maintained by protein synthesis and protein degradation. For protein degradation, the ubiquitin-proteasome pathway (UPP) plays an important role in the clearance of abnormal proteins, including misfolded, denatured or otherwise damaged proteins, or some short-lived regulatory proteins [3, 4]. The proteasome is a kind of highly conserved multi-subunit complex composed of a 20S core particle (CP) and two 19S regulatory particles (RP) in eukaryotes [5, 6]. The core particle is divided into two peripheral α rings and two central β rings, arranged as α1-7β1-7β1-7α1-7 configuration [4, 7]. X-ray crystallographic analyses indicated that there are three protease active sites (β1, β2, β5) in the proteasomal beta subunits. Depending on their N-terminal threonine residue hydrolysis, the enzyme centers of β1, β2 and β5 subunits were termed post-glutamyl peptide hydrolase-like (PGPH), trypsin-like and chymotrypsin-like (CT-L), respectively [8, 9]. Based on its complex structure, proteasome plays a variety of functions via the ubiquitin-proteasome pathway and has drawn much attention [10, 11].

In some protozoan parasites, proteasome was regarded as an attractive new chemotherapeutic target as it plays a key role in parasite biology and virulence [12, 13]. For example, inhibition of proteasomal function could retard morphological differentiation in specific stages of *Trypanosoma*, *Plasmodium* and *Entamoeba* and replication of *Plasmodium*, *Toxoplasma*, *Leishmania* and *Trypanosoma* [12]. It is also interesting to find that artemisinin and its derivatives, the most widely-used compounds against malaria, were found to kill malaria parasites by compromising parasite proteasome function and damaging protein [14, 15]. In *Trichomonas vaginalis*, proteomic analysis confirmed that this parasitic protozoan has all seven α and seven β subunits of the eukaryotic proteasome, among which, the activity of β1 and β5 can be inhibited by an anticancer proteasome inhibitor carmaphycin-17 [16]. In addition to parasitic protozoans, proteasomes are also fundamental to successful parasitism of the schistosome flatworm parasites. In *Schistosoma mansoni*, an endogenous proteasome inhibitor PI31 participated in the regulation of ubiquitin-proteasome pathway to maintain the stability of cells [17], and a peptide epoxyketone inhibitor derived from the marine natural product carmaphycin B (1 μM) decreased both worm motility and endogenous 20S proteasome activity, suggesting that 20S proteasome is involved in the regulation of worm motility [18].

Although the proteasome has received much attention as a potential chemotherapeutic target for treatment of a variety of infectious diseases caused by parasitic protozoans and trematodes, few studies have been done on parasitic nematodes. Fortunately, proteasome functions, especially those involved in worm development and life span, have been studied deeply in the free-living nematode *Caenorhabditis elegans* [1, 19–25]. The results show that the life span extension in *C. elegans* is dependent on the transcriptional activity of dauer formation abnormal/forkhead box class O (DAF-16/FOXO), skinhead-1 (SKN-1) and heat shock factor-1 (HSF-1) factors through regulation of downstream longevity genes, and the proteasomal β5 subunit (pbs-5) acts as a complex among DAF-16/FOXO, SKN-1, HSF-1 factors and their downstream targets [1]. Despite the functional importance of *pbs-5*, nothing is known about parasitic nematodes including the blood-feeding species *Haemonchus contortus*, even though a ubiquitination pathway model for *H. contortus* has been constructed [26]. This parasite is an important ruminant gastrointestinal parasitic nematode with a worldwide distribution, and its control mainly depends on anthelmintics. However, drug resistance has become a serious problem [27, 28]. Therefore, there is an urgent need to develop new drugs and vaccines. Understanding the mechanism of development and life span of *H. contortus* at the molecular level can help us to develop new drugs and discover potential vaccine candidates to control hemonchosis. In the present study, the proteasomal β5 subunit encoding gene (*Hc-pbs-5*) of *H. contortus* was isolated and characterized. The transcriptional pattern of *Hc-pbs-5* in eight developmental stages of *H. contortus* and the localization of *Hc*-PBS-5 in *H. contortus* adult worms were analyzed. In addition, a specific proteasome inhibitor bortezomib (BTZ) and RNA interference (RNAi) technique were employed to explore the role of *Hc-pbs-5* in the regulation of *H. contortus* developmental processes.

## Methods

### Ethics statement

All of the experimental animals used in this study were maintained in accordance with protocols approved by Animal Ethics Guidelines from the People’s Republic of China and the Scientific Ethic Committee of Huazhong Agricultural University (permit code: HZAUGO-2016–007).

### Maintenance of *Haemonchus contortus*

The Haecon 5 strain of *H. contortus* was maintained in goats (helminth-free), which were inoculated orally with 8000 infective third-stage (L3) larvae. Eggs were isolated from the feces of infected goats, and the larvae in free-living stages (L1s, L2s and L3s) were collected by copro-culture using the methods described previously [29]. Fourth-stage larvae (L4s) and adults of *H. contortus* were collected from the fourth stomach of the infected goats, which were euthanized by intravenous injection of pentobarbitone sodium (Lethobarb, Virbac Pty Ltd., Australia) at 8 or 30 days of infection, respectively. Then, the worms of the two parasitic stages were washed extensively in physiological saline to remove debris, and males and females were separated before freezing. The worms in each developmental stage were snap-frozen in liquid nitrogen and subsequently stored at − 80 °C until use.

### Isolation of the full-length cDNA of *Hc-pbs-5*

Total RNA was isolated from adult worms of *H. contortus* using TRIzol (Life Technologies, USA) following the manufacturer’s instructions, and then electrophoresis and spectrophotometry (Nano Drop Technologies, USA) were carried out to verify the RNA integrity and yields, respectively. Complementary DNA (cDNA) was synthesized from RNA (1 μg) using the RevertAid First Strand cDNA Synthesis Kit (Thermo Scientific). Based on the transcriptomic and genomic datasets for *H. contortus* (GenBank number *Hc-pbs-5*: HF965960.1) [30, 31] and two primers, Hc-pbs-5-cF and Hc-pbs-5-cR (Additional file 1: Table S1), were designed and used in PCR to obtain the full-length cDNA of *Hc-pbs-5* following previously described PCR procedures [32]. The amplified cDNA fragment was inserted into the pMD-19 T and directly sequenced in both directions (via TsingKe Biological Technology, Wuhan, China).

### Bioinformatic analyses

According to the full-length cDNA sequence of *Hc-pbs-5*, the amino acid sequence of *Hc-*PBS-5 was predicted using the software DNAstar (http://www.dnastar.com/). The cDNA sequence of *Hc-pbs-5* was compared with sequences in non-redundant databases using the BLASTx from the National Center for Biotechnology Information (NCBI) (http://www.ncbi.nlm.nih.gov/BLAST) to confirm its identity. For phylogenetic analysis, the predicted amino acid sequences of *Hc*-PBS-5 and 14 other homologs (Additional file 1: Table S1) were aligned using the software MEGA v.6.0 [33]. Homologous sequences from 14 species were retrieved from protein databases of NCBI (https://www.ncbi.nlm.nih.gov/protein), representing *Brugia malayi* (XP_001902498.1) [34], *Caenorhabditis briggsae* (CAP36972.1) [35], *Caenorhabditis elegans*(CAB04567.1) [36], *Caenorhabditis remanei* (EFO83800.1) [37], *Danio rerio* (AAH71478.1) [38], *Dictyocaulus viviparous* (KJH44249.1) [39], *Diploscapter pachys* (PAV66776.1) [40], *Drosophila melanogaster* (AAF58748.1) [41], *Homo sapiens* (NP_002788.1) [42], *Mus musculus* (NP_035316.1) [43], *Necator americanus* (ETN68175.1) [44], *Saccharomyces cerevisiae* (AAA34906.1) [45], *Toxocara canis* (KHN76925.1) [46] and *Xenopus laevis* (XP_018114995.1) [47]. The *S. cerevisiae* proteasome core particle subunit beta 1 (NP_012533.1) [48] was set as an outgroup. Phylogenetic analyses of aligned sequence data were conducted using the neighbor-joining (NJ), maximum parsimony (MP) and maximum likelihood (ML) methods employing the Jones-Taylor-Thornton (JTT) model; confidence limits were assessed using a bootstrap procedure employing 1000 pseudo-replicates [33].

The predicted amino acid sequence of *Hc*-PBS-5 and a series of reference sequences (Additional file 1: Table S1) were aligned using the software BioEdit (https://bioedit.software.informer.com) and the alignment adjusted manually. The three-dimensional structure of *S. cerevisiae* PBS-5 [5] was used to create homology model for that of *Hc-*PBS-5 using the SWISS-MODEL server (https://swissmodel.ex-pasy.org/). The protease active cores and binding sites were identified with reference to *S. cerevisiae* PBS-5 [8] and highlighted using the software Adobe Photoshop CS6.

### Transcriptional analysis using real-time PCR

The relative abundance of *Hc-pbs-5* was assessed in eight developmental stages (eggs, L1s, L2s, L3s, male L4s, female L4s, adult males and adult females) of *H. contortus* (Haecon-5 strain) as described previously [49]. First, the total RNA was extracted from the eight developmental stages of *H. contortus* using TRIzol (Life Technologies, USA), respectively, and the extracted RNA integrity and yields were verified by electrophoresis and spectrophotometry (Nano Drop Technologies, USA). Complementary DNA (cDNA) was reverse transcribed with 1 μg RNA by PrimeScript™ RT Reagent Kit with gDNA Eraser (Perfect Real Time) (Takara, Japan). The transcriptional level of *Hc-pbs-5* was detected by real-time PCR using the primers *Hc-pbs-5*-rtF/R (Additional file 1: Table S2) under the following protocol: 95 °C for 30 s, followed by 40 cycles at 95 °C for 15 s, 60 °C for 15 s and 72 °C for 20 s. The gene β-tubulin 8–9 of *H. contortus* was used as a normalizer with the specific primers Hc-tub8-9-rtF/R (Additional file 1: Table S2) for each of the triplicate samples [50]. An established formula was used to calculate the efficiency of the primers [51], and the relative quantities of egg (egg = 1) were used as a reference to analyze the data of the real-time PCR by the 2^−△△Ct^ method [51]. Transcriptional levels of *Hc-pbs-5* were assessed three times for each developmental stage of *H. contortus* included.

### Expression patterns by immunohistochemistry in adult *H. contortus*

Total RNA was isolated from adult worms of *H. contortus* using TRIzol (Life Technologies, USA) following the manufacturer’s instructions, and then electrophoresis and spectrophotometry (Nano Drop Technologies, USA) were carried out to verify the RNA integrity and yields, respectively; 1 μg RNA was reverse transcribed to cDNA by RevertAid First-Strand cDNA Synthesis Kit (Thermo Scientific, USA). One set of primers Hc-pbs-5-eF/eR (Additional file 1: Table S2) were designed to amplify the coding sequence of the protein *Hc-*PBS-5 from cDNA under the conditions: 95 °C for 3 min, followed by 35 cycles at 94 °C for 15 s, 55 °C for 15 s and 72 °C for 1 min, then 72 °C for 5 min. The amplicon was inserted into the expression vector pE-SUMO by a ClonExpressTM II One Step Cloning Kit (Vazyme Biotech Co., Ltd), which was transformed into BL21 (DE3) cells of *Escherichia coli*, followed by 1 mM isopropyl β-D-1-thiogalactopyranoside (IPTG) induction at 37 °C for 3 h to produce recombinant r*Hc*-PBS-5. Next, total protein was denaturated followed by renaturation to purified the recombinant r*Hc*-PBS-5. Then, purified r*Hc*-PBS-5 was concentrated using hyperspeed centrifugation method and analyzed by SDS-PAGE. The protein was inoculated subcutaneously into rabbits to produce the polyclonal antibody of *Hc-*PBS-5 (4 immunizations, 5 weeks apart). A pre-bleed was taken from each rabbit prior to first injection while a final bleed was taken 1 week after the last immunization. The serum from the pre-bleed was designated as negative serum while the serum from the final bleed was designated as polyclonal anti-*Hc-*PBS-5 antibody (positive serum). All sera were analyzed by Western blot using the total protein of *H. contortus* extracted from adult worms by using the Total Protein Extraction Kit (Bestbio Company, China).

Using the serum, the expression pattern of *Hc-*PBS-5 was detected in adult males and females of *H. contortus* by immunohistochemistry. Approximately 50 *H. contortus* adult males or females were fixed in 4% paraformaldehyde (Biosharp, China) at 4 °C for 3 days, respectively. Each single worm from the paraformaldehyde was dehydrated in a series of graded ethanol (75% for 4 h, 85% for 2 h, 90% for 2 h, 95% for 1 h each and 100% two times for 30 min) sequentially, and embedded in paraffin. The single worm embedded in paraffin was cut into sections (4 μm) for flattening on polysine slides, then paraffined in xylene (two times for 20 min) and rehydrated in a series of graded ethanol (100% two times for 10 min; 95% one time for 5 min, 90% one time for 5 min, 80% one time for 5 min, 70% one time for 5 min each), followed by washing with phosphate buffer solution (PBS, three times for 5 min). The microwave was used to recover the antigens, and 3% hydrogen peroxide was used to eliminate the endogenous catalase. After washing with PBS for three times (5 min), the sections were blocked with 5% bovine serum albumin (BSA) for 20 min in a humidified chamber and then incubated with approximately 50 μl polyclonal anti-*Hc*-PBS-5 antibody (positive serum) or negative serum (each at 1:100 dilution) at 4 °C overnight, respectively. The serum was washed off with PBS, and the sections were incubated with the anti-rabbit immunoglobulin (IgG) (raised in sheep) conjugated with fluorescein (Aspen, China) at 37 °C for 50 min in a dark place. After washing off the secondary antibody, the sections were incubated at room temperature for 5 min with 4, 6-diamidino-2-phenylindole (DAPI) solution in a dark place. The sections were washed in PBS three times (5 min) again and then assessed in detail using an epifluorescence microscope (Olympus CX-21, Japan). All images were processed using Photoshop CS 6.0.

### Assessment of the chymotrypsin-like activity of a mixture native *Hc*-PBS-5 by the specific proteasome inhibitor bortezomib (BTZ)

Bortezomib (BTZ), a specific proteasome inhibitor, has high affinity, specificity and selectivity to the enzyme activity of proteasomal β5 subunit (chymotrypsin-like) [52, 53]. The substrate N-succinyl-Leu-Leu-Val-Tyr-7-amino-4-methylcoumarin (SLLVT-AMC) can be cleaved specifically by proteasomal β5 subunit (chymotrypsin-like) and cleaved substrate can show the fluorescent [52]. In this study, the whole worm native protein was extracted from *H. contortus* xL3s. First, the L3s of *H. contortus* collected from fecal cultures were treated by 0.1% sodium hypochlorite/PBS for 30 min at 38 °C to exsheath in vitro, and then the exsheathed L3s (xL3s) were washed four times with PBS by centrifugation at 600 g (5 min) at 23 °C. After the final washing, xL3s were suspended in 2 ml solution with 1 M Tris-HCl (pH 7.4), 1 mM EDTA, 2 mM ATP, 4 mM DTT and 20% glycerinum. The mixture was broken by ultrasound for 10 min at 4 °C and transferred to a clean homogenizer for grinding for at least 30 min (on ice). Centrifuged at 10,000 g (5 min) at 23 °C, supernatant was used to analyze the concentration and quality by Bradford Protein Assay Kit (Beyotime, China) and SDS-PAGE, respectively. For the mixture of proteins from the soluble fraction of the *H. contortus* extract (native *Hc*-PBS-5 included), 150 μg/well was added into 96-well plates. The inhibitor BTZ was set as different final concentrations (0.001 μM, 0.01 μM, 0.1 μM, 1.0 μM; in triplicate) to be added into the wells with a mixture of native *Hc*-PBS-5 protein. DMSO (in triplicate) was set as a control group. The substrate SLLVT-AMC was added, and the final concentration of SLLVT-AMC was 40 μM. After incubation at 37 °C for 1 h, the fluorescence of the culture was analyzed using an Infinite F200 multi-mode microplate reader (Biotek, USA), with excitation and emission wavelength 380 nm and 460 nm, respectively.

### Effect of bortezomib (BTZ) on egg hatching and development of xL3 in* H. contortus*

Eggs of *H. contortus* were isolated from the overnight collected feces of infected goats using the methods described previously [29] and counted in physiological saline solution (PS). The culture medium (CM) of *H. contortus* eggs was optimized as previously described [29], which contained 20% Nutrient Medium (Earle’s Balanced Salt Solution and 1% yeast extract in sterile PS), 17% eggs in PS and 63% sterile PS. The final concentration of eggs was 5–10 eggs/μl. Each culture flask (12.5 cm^2^) could be filled with 2.5 ml CM and different concentrations of inhibitor BTZ. The final concentrations of BTZ were set as 0.5 μM, 1 μM, 1.5 μM and 2 μM (in triplicate). DMSO (in triplicate) was set as a control group. The culture flasks were sealed and incubated at 28 °C. The hatching rates of eggs were detected after incubation for 19 h, 23 h and 28 h, respectively.

The L3s of *H. contortus* from fecal cultures were treated by 0.1% sodium hypochlorite/PS for 30 min at 38 °C to exsheath in vitro, and then the exsheathed L3s (xL3s) were washed twice with sterile PS and four times with another sterile PS containing antibiotic–antimycotic solution (Gibco, USA) by centrifugation at 600 g (5 min) at 23 °C (Gibco, USA). After the final washing, xL3s were suspended in Earle’s Balanced Salt Solution (EBSS, Sigma, USA; pH adjusted to 5.2) with antibiotic-antimycotic solution (Gibco, USA); 100 μL of EBSS cultures containing xL3s and different concentrations of inhibitor BTZ were added into 96-well plates. The final concentration of xL3s was 5–10 xL3/μl, and the final concentrations of BTZ were set as 0.01 μM, 0.1 μM, 1 μM and 10 μM (in triplicate). DMSO (in triplicate) was set as a control group. After incubation at 37 °C in 20% CO_2_ for 7 days, the numbers of L3s and L4s would be counted according to the morphological changes of the buccal capsule by microscopy [32, 54, 55].

### RNA interference (RNAi) by soaking in *H. contortus* L3s

The double-stranded RNA for soaking was synthesized as previously described [32, 49, 56]. In brief, the coding sequence of the proposed functional domain—*Hc*-PBS-5 (618 bp)—was amplified by two sets of specific primers (*Hc-pbs-5*-sF1/sR1 and Hc-pbs-5-sF2/sR2, Additional file 1: Table S2) for constructing two plasmids. One set of specific primers (Hc-pbs-5-sF1/sR1) was tagged with a T7 promoter site in the forward direction and a restriction enzyme BamH I site in the reverse direction, respectively, which was used to amplify the template that produced the antisense single-stranded RNA (antisense ssRNA). The other set of specific primers (Hc-pbs-5-sF2/sR2) was tagged with restriction enzyme BamH I site in the forward and a T7 promoter site in the reverse direction, respectively, which was used to amplify the template that produced the sense single-stranded RNA (sense ssRNA). The procedure for amplification was as follows: 95 °C/3 min, followed by 94 °C/30 s, 55 °C/30 s; 72 °C/1 min for 35 cycles; 72 °C/5 min. Then, the two amplicons were inserted into the pTOPO-Blunt Simple vector (Aidlab Biotechnologies Co., Ltd.) using the ClonExpressTM II One Step Cloning Kit (Vazyme Biotech Co., Ltd.), respectively. A *cry1Ac* gene from *Bacillus thuringiensis* (*Bt-cry1Ac*, GenBank accession no. GU322939.1) was used as an irrelevant control in the present assay [31]. All plasmids were extracted using the plasmid Maxi Kit (Aidlab Biotechnologies Co., Ltd.), respectively, and their yields were verified by spectrophotometry (Nano Drop Technologies, USA), respectively. The extracted plasmids were stored at -20 °C until use. Each template of *Hc-pbs-5* or *Bt-cry1Ac* fragments, linearized by the restriction enzyme Hind III or BamH I, respectively, was used to synthesize single-stranded RNA (ssRNA) by RNA large-scale T7 production system according to the instruction manual of MEGAscript® T7 Transcription Kit (Ambion, USA), respectively. Equal amounts (500 µg) of sense ssRNA and antisense ssRNA were used to synthesize double-stranded RNA (dsRNA) using the manufacturer’s protocol (Ambion, USA). The yields and quality of linearized templates, ssRNAs and dsRNA were verified by spectrophotometry (NanoDrop Technologies) and electrophoresis, respectively. All RNA samples were frozen immediately and stored at − 80 °C until use.

The RNA interference by soaking was performed as previously described [32]. The L3s of *H. contortus* from fecal cultures were treated by 0.1% sodium hypochlorite/PBS for 30 min at 38 °C to exsheath in vitro, and then the exsheathed L3s (xL3s) were washed twice with sterile PBS and four times with another sterile PBS containing antibiotic-antimycotic solution (Gibco, USA) by centrifugation at 600 g (5 min) at 23 °C. After the final washing, xL3s were suspended in Earle’s Balanced Salt Solution (EBSS, Sigma, USA; pH adjusted to 5.2) with antibiotic-antimycotic solution (Gibco, USA). The *Hc-pbs-5*-specfic dsRNA, Bt-cry1Ac-specific dsRNA (irrelevant control) and nuclease-free water (untreated control) were pre-incubated (separately) with RNasin (8 U) and Lipofectin Reagent (Invitrogen, USA) for 10 min at 25 °C (room temperature), respectively, and then added to 30 μL of EBSS cultures containing xL3s. The final concentration of xL3s was 33 xL3/μl, and the final concentration of dsRNA was 1 mg/ml. The culture was incubated at 37 °C in 20% CO_2_ for 24 h, and then 300 larvae were transferred to 100 μl of new EBSS (in triplicate) to incubate for another 7 days to count the numbers of L3s and L4s according to the morphological changes of the buccal capsule by microscopy [32, 54, 55]. The remaining larvae were collected to extract total RNA for detecting the transcriptional changes of the gene *Hc-pbs-5* by real-time PCR with one set of primer *Hc-pbs-5*-rtF/R (Additional file 1: Table S2) under the conditions: 95 °C for 30 s, followed by 40 cycles at 95 °C for 15 s, 60 °C for 15 s and 72 °C for 20 s. The 18S ssrRNA was used as a reference marker [57], and information on primers Hc-18 s-rtF/R was shown in Additional file 1: Table S2. The method for calculation was performed as previously described in the present study.

### Data presentation and analyses

All data were shown as means ± standard error of the mean (SEM), and Tukey’s multiple comparisons test was carried out. A one-way ANOVA was conducted for comparing the transcriptional analysis of *Hc-pbs-5* in different developmental stages and the transcriptional changes of *Hc-pbs-5* after soaking dsRNA for 24 h. The rest of comparisons in the present study were conducted by two-way ANOVA. *P* < 0.05 was considered statistically significant (*). *P* < 0.01 and *P* < 0.001 were considered as highly statistically significant (** and ***, respectively). Statistical differences at 0.05 level were represented by lowercase letters for the transcriptional analysis of *Hc-pbs-5* in different developmental stages. Graphs were produced using the program GraphPad Prism 6 and processed using Photoshop CS 6.0.

## Results

### Characterization of *Hc-pbs-5* gene from* H. contortus*

**Fig. 1:**
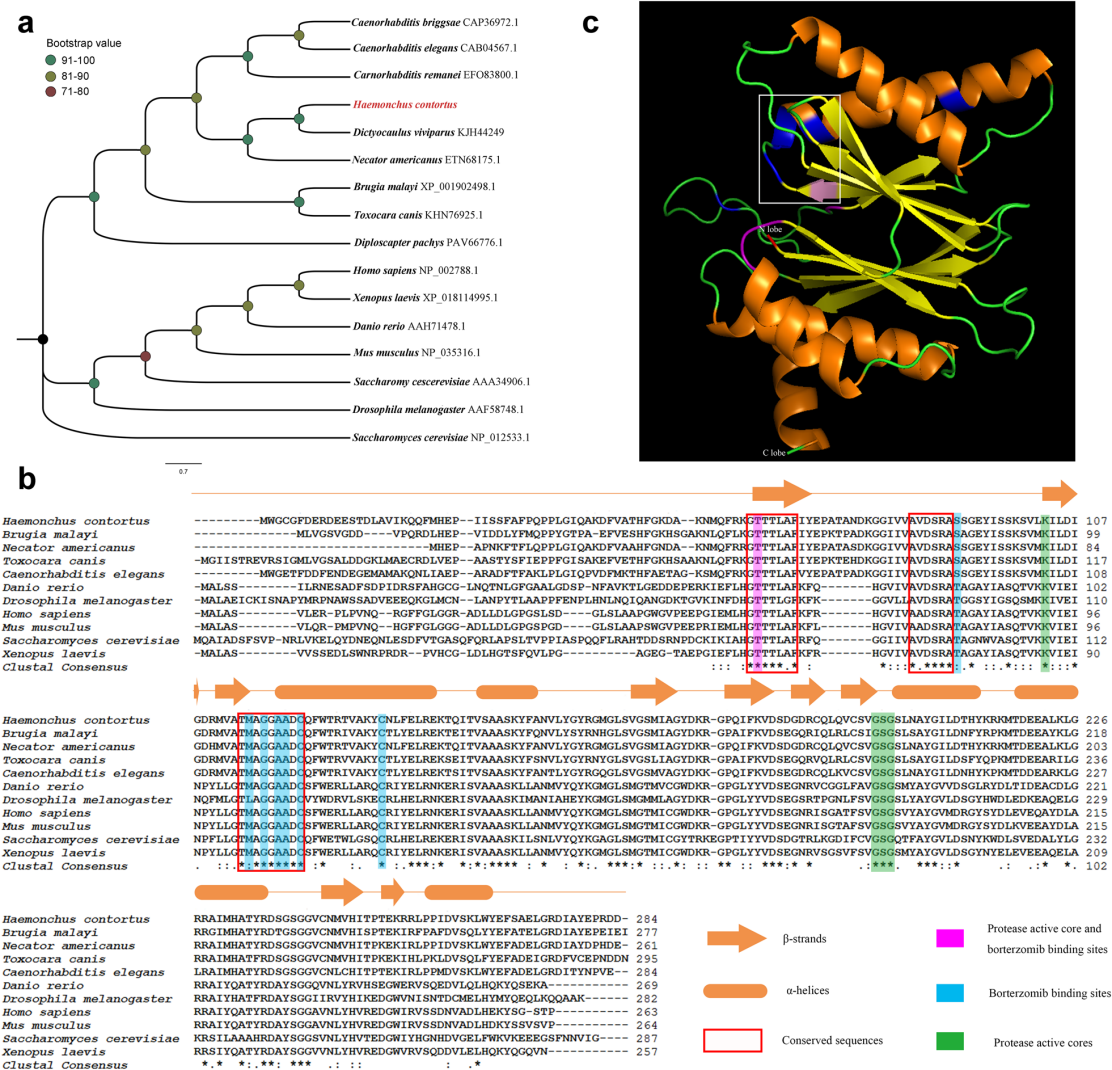
*Hc*-PBS-5 displays conserved features with PBS-5 homologs from selected species. **a** The rooted neighbor-joining tree showing the phylogenetic relationships of *Haemonchus contortus* proteasomal β5 subunit (*Hc*-PBS-5) with other proteasomal β5 subunit homologs, whose sequences are listed in Additional file 1: Table S1. The *Saccharomyces cerevisiae* proteasome core particle subunit beta 1 was set as an outgroup. Their corresponding accession numbers are given to the right of each species name. The tree was built employing the Jones-Taylor-Thornton (JTT) model, and confidence limits were assessed using a bootstrap procedure employing 1000 pseudo-replicates. Nodal support values for each clade are color coded. **b** Amino acid sequence alignment of *Hc*-PBS-5 and other selected species, whose sequences are listed in Additional file 1: Table S1. Secondary structure elements were identified with reference to *Saccharomyces cerevisiae* PBS-5 and labeled above the sequence with α-helices represented with orange cylinders and β-strands represented with orange arrows. Conserved sequences were indicated in red squares; protease active core and bortezomib (BTZ) binding sites in purple and BTZ binding site in cyan and protease active cores in green. (**c**) Putative three-dimensional structure of *Hc*-PBS-5 was generated using the SWISS-MODEL server. In the putative three-dimensional structure, α helixes are marked in orange, β strands are in yellow, and the S1 (‘specificity’) pocket is marked with a white box. Important amino acids are also color coded: protease active core and bortezomib (BTZ) binding site Thr64 is in red, Met115 located in the bottle of the specific S1 pocket is in pink, BTZ binding sites are in purple, and protease active cores are in blue

The coding sequence of *Hc-pbs-5* verified in the present study (GenBank accession no. OP168759) was 855 bp long, encoding 284 amino acids (aa). The predicted protein sequence of *Hc-PBS-5* from *H. contortus* was aligned with other proteasomal β5 subunits (PBS-5) from 14 species represented and subjected to phylogenetic analyses (Fig. 1a). *Hc-PBS-5* had the closest relationship with PBS-5 of *N. americanus* (70% nodal support), which grouped together with PBS-5 of *D. viviparus* (98 nodal support). These three PBS-5s grouped together with PBS-5 homologs of other nematodes represented (100 nodal support) to the exclusion of PBS-5 homologs from six non-nematodes represented.

The amino acid sequence of *Hc*-PBS-5 had a relatively high range of similarities (34.5–84.8%) to homologs of various organisms including *N. americanus* (84.8%), *C. elegans* (77.1%), *B. malayi* (70.3%), *T. canis* (68.4%), *D. melanogaster* (38%), *X. laevis* (37.3%), *D. rerio* (35.9%), *M. musculus* (35.6%), *H. sapiens* (35.2%) and *S. cerevisiae* (34.5%). The alignment of these sequences indicated that *Hc*-PBS-5 had core conservative sequences (65–250 aa), which belong to N-terminal nucleophile (Ntn) family of hydrolases. In these core conservative sequences, there were five chymotrypsin active core sites (Thr68, Lys103, Gly199, Ser200 and Gly201) and eight BTZ binding sites (Thr68, Ser91, Met115, Gly117, Ala119, Ala120, Cys122 and Cys133) [8] (Fig. 1b). The amino acids at all other sites were conserved in the homologs of organisms represented, except for Ser91 in nematodes, which was different from Thr in vertebrates and yeast. DISPHOS [58] was used to predict the possible protein phosphorylation sites, and nine possible sites were found in *Hc*-PBS-5, which were Ser88, Ser91, Ser92, Vla95, Vla206, Val235, Thr252, Vla267 and Vla279. Except for phosphorylation sites, modification sites for proteins also include glycosylation sites. The NetOGlyc 3.1 tool was used to predict potential glycosylation sites in the protein; however, no potential glycosylation sites were identified based on a score (> 0.5). The putative three-dimensional structure of *Hc*-PBS-5 protein showed a typical "sandwich" shape with unparallel β-strands in the middle and α-helical peptides on both sides (Fig. 1c). The active site Thr68 is close to the specific S1 pocket, and BTZ-binding site Met115 is located in the bottle of the specific S1 (‘specificity’) pocket.

### Transcriptional level of *Hc-pbs-5* throughout the lifespan of *H. contortus*

**Fig. 2:**
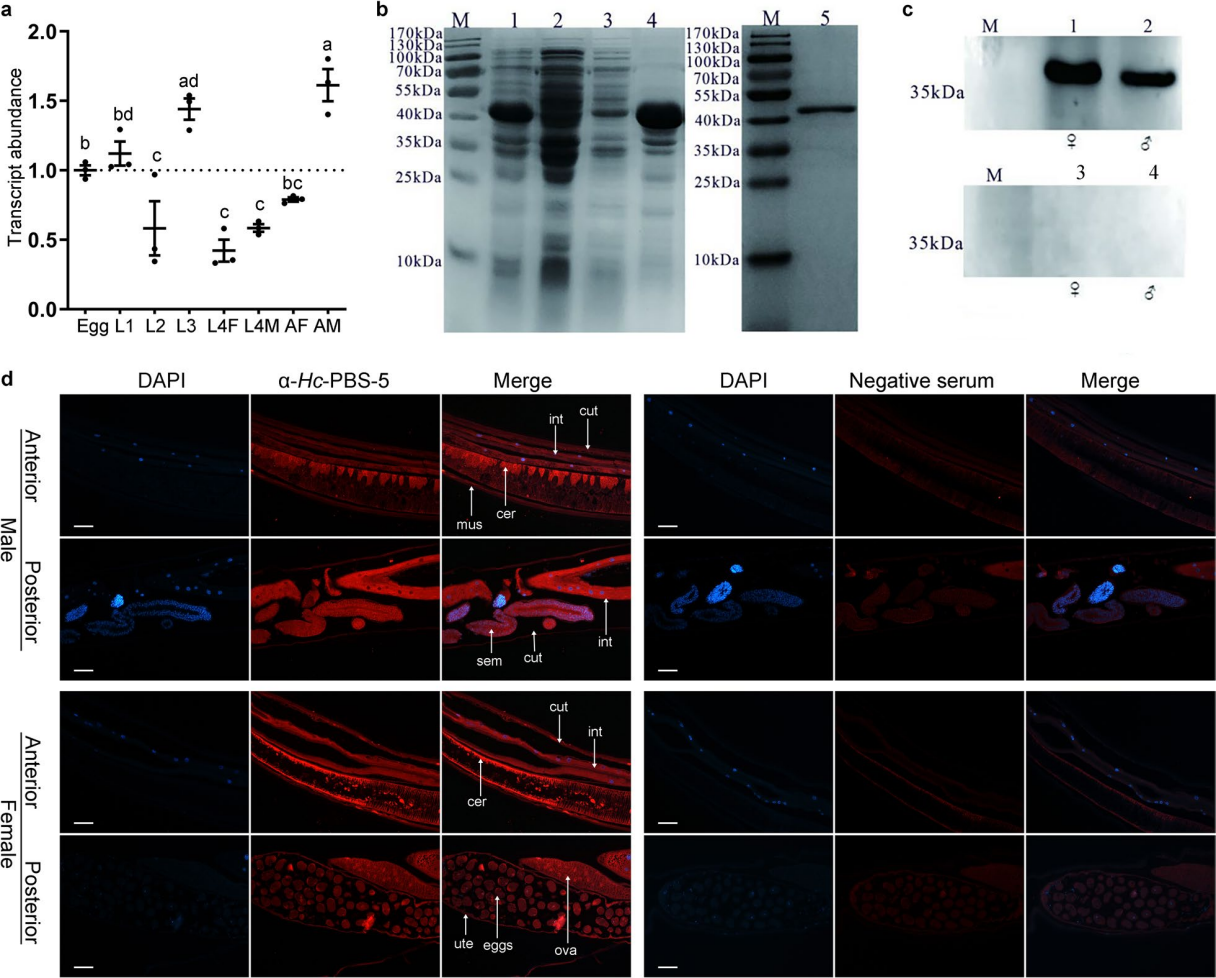
Spatiotemporal expression of *Hc-*PBS-5 in *Haemonchus contortus*. **a** The relative quantities of *Hc-pbs-5* was assessed by real-time PCR in eight developmental stages of *H. contortus*. The relative quantities (compared with egg, egg = 1) are shown as means ± standard error of the mean (SEM). Statistical difference at 0.05 level was represented by lowercase letters for the transcriptional analysis of *Hc-pbs-5* in different developmental stages, the different lowercase letters (a, b, c, d) mean *P* < 0.05 while the same lowercase letters mean *P* > 0.05. **b** SDS-PAGE analyses of recombinant *Hc-*PBS-5. M, protein marker; lane 1–2, recombinant *Hc*-PBS-5 was expressed with and without 1 mM isopropyl-β-d-thiogalactoside (IPTG) for 3 h; lane 3, the supernatant and the inclusion body of recombinant *Hc-*PBS-5 induced expression; lane 5, the purified recombinant *Hc-*PBS-5. **c** Western blot analysis using total protein from adult *H. contortus* worms. M, protein marker; lane 1–2, detection of *Hc-*PBS-5 by the rabbit antiserum containing anti-r*Hc-*PBS-5 antibody from the final bleed (positive serum); lane 3–4, the same blot re-probed with pro-immune serum (negative control); ♀, adult females of *H. contortus*; ♂, adult males of *H. contortus*. **d** The localization of *Hc-*PBS-5 in *H. contortus* adult males and adult females by the serum from the final bleed that contained polyclonal anti-*Hc*-PBS-5 antibody (positive serum) and negative serum, respectively. No fluorescence labeling was observed in negative controls probed with negative serum. int, intestine; cut, outer cuticle; mus, muscle cells under the outer cuticle; cer, cervical glands; sem, seminal vesicle. ute, uterine wall; eggs, eggs in uterus; ova, ovarian. Each single image was amplified 100 ×, scale bars: 100 μm

Real-time PCR results suggested that *Hc-pbs-5* was transcribed at the highest level in L3s and adult males of *H. contortus*, and there was no difference between these two developmental stages. The relative transcriptions of *Hc-pbs-5* in L2s, female L4s and male L4s were lowest and showed significant differences compared with the relative transcription in eggs, L1s, L3s (ANOVA, *F*_(7,16)_ = 20, *P* < 0.001) and adult males (*P* < 0.001) (Fig. 2a). However, there was no difference in the relative transcription of *Hc-pbs-5* among L2s, female L4s or male L4s (Fig. 2a). The relative transcriptions of *Hc-pbs-5* in eggs, L1s and adult females were at a middle level. The relative transcriptions of *Hc-pbs-5* in eggs and L1s have significant difference compared with the relative transcription in L2s (*P* = 0.0015 and *P* < 0.001), female L4s (*P* < 0.001 and *P* < 0.001), male L4s (*P* = 0.0201 and *P* = 0.0018) and adult males (*P* < 0.001 and *P* = 0.0043), respectively (Fig. 2a). The relative transcription of *Hc-pbs-5* in adult females was significantly lower than that in L3s (*P* < 0.001) and adult males (P < 0.001) (Fig. 2a).

### Localization of Hc-PBS-5 in adult *H. contortus* by immunofluorescence

The prokaryotic expression protein, *Hc-*PBS-5 (284 aa, together with a SUMO tag, ~ 43.2 kDa), was purified and identified, as shown in Fig. 2b, and its rabbit-derived polyclonal antibody was produced. We observed that positive-serum antibody could bind specifically to the native *Hc*-PBS-5 (~ 34.8 kDa) from both sexes of adult *H. contortus*, whereas the negative-serum antibody did not recognize any *H. contortus* proteins (Fig. 2c). Then, the expression pattern of *Hc*-PBS-5 in adult *H. contortus* was detected by this polyclonal serum antibody. In adult male worms, *Hc*-PBS-5 was mainly located in the intestine, outer cuticle, muscle cells under the outer cuticle, cervical glands and seminal vesicles (Fig. 2d). In adult female worms, *Hc*-PBS-5 was mainly located in the intestine, outer cuticle, cervical glands, uterine wall, eggs and ovary (Fig. 2d).

### Bortezomib (BTZ) inhibits the enzyme activity of proteasomal β5 subunit (chymotrypsin-like) of* H. contortus*

**Fig. 3:**
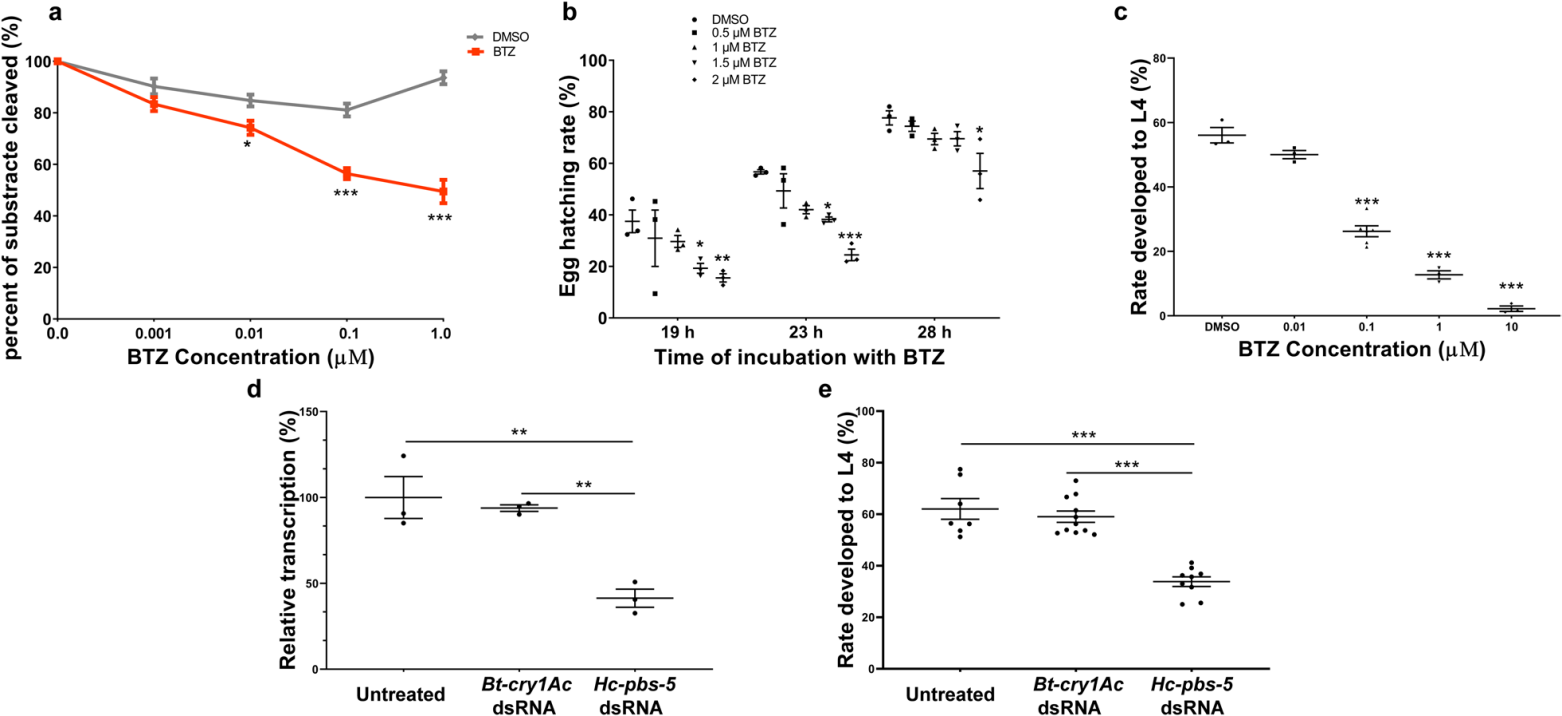
*Hc*-PBS-5 is involved in regulating the egg hatching and larval development of *Haemonchus contortus*. **a** The effect of BTZ on the chymotrypsin-like activity of whole protein containing native *Hc*-PBS-5. **b** The effect of BTZ on egg hatching by soaking with different concentrations (0.5 μM, 1 μM, 1.5 μM, 2 μM) of BTZ for different times. **c** The effect of BTZ on the developmental rate from xL3s to L4s in vitro by soaking with different concentrations (0.01 μM, 0.1 μM, 1 μM, 10 μM) of BTZ for 7 days. **d** Relative transcript changes of *Hc-pbs-5* after RNAi detected by real-time PCR; **e** rate of L4s developed from xL3s in vitro for 7 days after RNAi. *P* < 0.05 was considered as statistically significant (*). *P* < 0.01 and *P* < 0.001 were considered as highly statistically significant (** and ***, respectively)

According to the alignment of proteasomal β5 subunit among 11 species represented, we found *Hc*-PBS-5 containing eight BTZ binding sites (Fig. 1b), so whole protein containing native protein *Hc*-PBS-5 was extracted from *H. contortus* xL3s, and then the inhibitor bortezomib (BTZ) was used to assess the chymotrypsin-like activity of *Hc*-PBS-5. Compared with the control group, the chymotrypsin-like activity of *Hc*-PBS-5 decreased significantly with the concentration of BTZ at 0.01 μM (*F*_(4, 20)_ = 22.75, *P* = 0.0463), 0.1 μM (*P* < 0.001) and 1 μM (*P* < 0.001), but not at 0.001 μM (*P* = 0.3281) (Fig. 3a). The IC50 of BTZ in *H. contortus* L3 was calculated to be 0.6218 µM.

### Bortezomib (BTZ) inhibited egg hatching and exsheathed L3 to L4 development of *H. contortus*

The proportions of egg hatching of *H. contortus* were detected after incubation with different concentration of BTZ for 19 h, 23 h and 28 h, respectively. After incubation for 19 h, the proportions of egg hatching showed no difference between the control group, and treatment group with the final concentration of BTZ was at 0.5 µM or 1 µM, but the proportions of egg hatching showed significant decrease when the final concentration of BTZ went up to 1.5 µM (*F*_(8, 30)_ = 0.57, *P* = 0.0397) and 2 µM (*P* = 0.0087) (Fig. 3b). After incubation for 23 h, the proportions of egg hatching also showed no difference between the control group and the treatment group with the final concentration of BTZ at 0.5 µM or 1 µM, but the proportions of egg hatching showed a significant decrease when the final concentration of BTZ went up to 1.5 µM (*P* = 0.0359) and 2 µM (*P* < 0.001) (Fig. 3b). After incubation for 28 h, the proportions of egg hatching reached 80% in control group (Fig. 3b). Compared with the control group, the proportions of egg hatching showed no difference when the final concentration of BTZ at 0.5 µM, 1 µM and 1.5 µM (Fig. 3b). There was significant difference between the control group and treatment group when the final concentration of BTZ was at 2 µM (*P* = 0.0152) after incubation for 28 h (Fig. 3b).

The effect of BTZ on the development of *H. contortus* xL3s was detected in vitro. In vitro, the xL3s can develop to L4s at 37 °C in 20% CO_2_ for 7 days [31]. The results suggested that BTZ significantly inhibited xL3 development to L4 at 0.1 µM, (*F*_(4, 26)_ = 265.70, *P* < 0.001), 1 µM (*P* < 0.001) and 10 µM (*P* < 0.001). However, the proportions of L4s showed no difference between the control group and treatment group where the final concentration of BTZ was 0.01 µM (Fig. 3c).

### Specific *Hc-pbs-5* dsRNA inhibited the development of* H. contortus* xL3s in vitro

After soaking of xL3s in *Hc-pbs-5* dsRNA for 24 h, the transcription of *Hc-pbs-5* in the RNAi group decreased significantly compared with the untreated control, *F*_(2, 6)_ = 99.51 (no dsRNA, *P* = 0.0042) and irrelevant dsRNA control group (*Bt-cry1Ac* dsRNA, *P* = 0.0073) (Fig. 3d). There was no difference in the transcription of *Hc-pbs-5* between the untreated control and irrelevant dsRNA control group (Fig. 3d). After incubation for another 7 days, only about 30% *Hc-pbs-5* dsRNA-treated xL3s could develop to L4s with a mature mouth cavity, which was significantly less than that of the untreated control (*F*_(2, 6)_ = 99.51, *P* < 0.001) and irrelevant dsRNA control group (*P* < 0.001) (Fig. 3e). The proportion of larvae that developed to L4s showed no difference between the untreated control and irrelevant dsRNA control group (Fig. 3e).

## Discussion

### Conserved three-dimensional structure of *Hc*-PBS-5

Since the first purification of the proteasome 26S in 1980 [59], both 20S and 26S proteasomes have been purified continuously from a variety of tissue cells by different means and are classified as proteasome family because of the similar physical and biochemical properties for decades [60–63]. Subsequently, the crystal structures of proteasome were obtained successively in *Thermoplasma acidophilum* [64] and *Saccharomyces cerevisiae* [5], and the similar crystal structures from these two species suggested that the proteasome had high structural conservation. In the present study, phylogenetic analysis revealed that *Hc-PBS-5* and the homologs of the species represented grouped together (Fig. 1a), which was consistent with the alignment results where the amino acids of *Hc*-PBS-5 were highly conserved, especially in the core conservative sequences of Ntn family of hydrolases (65–250 aa in *Hc*-PBS-5, Fig. 1b). These results suggested that proteasomal β5 subunit was highly conserved across different species and had conserved function of protease catalytic activity. In the core conservative sequences of *Hc*-PBS-5, Thr68 was not only an important BTZ binding site but also a chymotrypsin active core site. The N-terminal threonine or serine of Ntn family of hydrolases could act as nucleophiles with substrates to catalyze hydrolysis [65] and threonine of processed β subunits is the core element of nucleophilic attack [5]. Although the various subunits of proteasome have active sites, but not all of the active sites can bind with β5 subunit inhibitors and hydrophobic amino acids specifically, because the binding is associated with the specificity of S1 pocket. The amino acid residue Met115 located in the bottle of the specific S1 pocket can determine the specificity of the chymotrypsin activity of proteasomal β5 subunit and help the proteasomal subunit select substrates [5]. In the putative three-dimensional structure of *Hc*-PBS-5, the active site Thr68 is close to the specific S1 pocket, and Met115 is located in the bottle of the specific S1 pocket, suggesting that *Hc*-PBS-5 had the specificity of the chymotrypsin activity and could bind BTZ specifically.

### *Hc*-PBS-5 functions in the growth, development and life span

Studies of proteasome in parasites have shown that proteasome may be involved in the growth and reproduction of parasites. Two proteasome inhibitors, MG-132 and proteasome inhibitor 1, were shown to block growth and intracellular development of *Toxoplasma gondii* [66]. In *Schistosoma mansoni*, knockdown of the expression of the proteasome subunit, SmRPN11/POH1, could decrease 78% of parasite viability [67]. In the present study, we found that *Hc*-PBS-5 was mainly located in the digestive and reproductive systems of *H. contortus* adult worms (Fig. 2d). In *C. elegans*, the digestive system was related with food intake, nutrient absorption, storage and metabolism (http://www.wormatlas.org/hermaphrodite/intestine/Inframeset.htlm) while the reproductive system was related to reproductive development, considering the close relationship between *Hc*-PBS-5 and *Ce*-PBS-5, from which it can speculated that the *Hc-pbs-5* might be involved in regulating the growth and reproduction of *H. contortus*. In the egg hatching assay, the inhibitor BTZ in the culture medium could enter into the eggs and bind to proteasomal β5 subunit to affect the development of the egg embryo cells and the results showed that relatively high concentration (1.5 µM and 2 µM) of BTZ could inhibit *H. contortus* egg hatching (Fig. 3b). Compared with the control group, the proportion of egg hatching for 19 h and 23 h significantly decreased when the concentrations of BTZ were 1.5 µM and 2 µM, respectively, suggesting that the inhibitor BTZ could slow down the egg embryo development during the hatching stage. It usually takes about 24 h for an *H. contortus* egg to develop into L1 at 28 °C [49]. Compared with the control group, the proportion of egg hatching significantly decreased when the eggs were incubated with 2 µM of BTZ for 28 h, but the proportion of egg hatching did not significantly decrease when the eggs were incubated with 1.5 µM of BTZ for 28 h. This result hinted that a high concentration (2 µM) of BTZ may cease the egg development and reduce the final proportion of egg hatching in *H. contortus*. The result of RNAi experiment also showed that the development of many xL3s slowed down as they were unable to develop to L4s, which supported our assumption that the *Hc-pbs-5* gene was involved in the growth and development of *H. contortus*.

For the regulation of proteasome gene transcription, as a transcription regulator of 26S proteasome, the *Saccharomyces cerevisiae* gene stress-regulated transcription factor (Rpn4) has been found to yield a negative feedback circuit [68] as Rpn4 becomes more stable and accumulates when the activity of the proteasome is impaired, such as cytotoxicity, which in turn promotes the transcription of the proteasome gene and accumulates the proteasome. When the activity of the proteasome is restored, the proteasome can degrade the transcription factor Rpn4, and then the transcriptions of proteasome genes were restored to basic levels [69]. Proteasome catalytic active site is mainly on the β subunits, but each β subunit cannot independently carry out hydrolysis enzyme activity; it must closely connect with other subunits to perform the functions [5, 64]. Expression of a single subunit gene neither detects the enzyme activity nor finds the monomer of this subunit in the cell [5, 64]. Therefore, it is speculated that there was another regulatory mechanism to regulate the proteasome activity. In human and mouse cells, overexpression of chymotrypsin activity could greatly increase proteasome activity by increasing the activity of all α and β subunits of the proteasome[70, 71], so chymotrypsin active site may be the rate-limiting site of action for degradation of substrate proteins. However, based on coordinated regulatory mechanisms of each subunit, the reduction of chymotrypsin activity is not directly related to the reduction of substrate protein decomposition, because inhibiting the activity of the active sites of β5 subunit can lead to increased expression and activity of other subunits [72], suggesting that there could be a compensatory mechanism among proteasome subunits, which means that the activity of a downregulated subunit should be supplemented by enhancing the activity of other subunits. In the inhibitor treatment assay, the proportion of egg hatching gradually decreased with the increase of BTZ concentration (1.5 μM and 2.0 μM) at the earlier stages of egg hatching (19 h and 23 h, respectively); however, the proportion of egg hatching did not significantly decrease in the later stage of egg hatching (28 h) when the concentration of BTZ was at 1.5 µM. It is speculated that there may be two reasons. One may be the involvement of transcriptional regulators in the negative feedback regulatory mechanism. The decrease of chymotrypsin activity could affect the growth and development of eggs, which would also destroy the internal environment of egg cells. With the extension of incubation time, the internal environment of egg cells will be continuously destroyed, which will gradually increase the amount of damaged proteasome activity and then lead to an increased amount of transcription factor Rpn4, which promotes proteasome transcription and expression, and finally the rescue of chymotrypsin activity. Another reason may be related to the compensatory regulation mechanism. When chymotrypsin activity was downregulated by 1.5 μM of BTZ, the activities of β1 and β2 subunits were enhanced so that the overall protease activity level remained stable. However, when the concentration of inhibitor reached 2.0 μM, it was far beyond the IC50 value of BTZ (0.6218 μM) and within the IC50 range of cancer cells [73]; the toxicity of inhibitor itself may cause death of eggs and reduce the proportion of egg hatching.

As the core subunit of the proteasome, β5 subunit plays an important role in regulating cell life cycle. Enhancement of proteasomal β5 subunit activity can prolong the life of human fibroblasts [74]; in addition, overexpression of *Ce-pbs-5* gene in *C. elegans* can prolong the lifespan of worms to a certain extent [74]. Does the silencing of *Hc-pbs-5* gene or inhibition of *Hc*-PBS-5 protein have a certain effect on the life span of *H. contortus*? In *S. cerevisiae*, the chymotrypsin activity could be irreversibly inhibited but not completely inactivated through the boronic acid-based proteasome inhibitor BTZ binding to the core particle of the proteasomal β5 subunit [8]. In our present study, we found that 2.0 μM of BTZ could significantly reduce the proportion of eggs developing to L1 stage; in vitro experiments on xL3 larval development showed that 0.1 μM, 1 μM and 10 μM of BTZ could reduce the proportion of xL3s developing to L4s to different degrees. All these results indicated that use of the inhibitor BTZ to inhibit the function of *Hc-*PBS-5 protein could affect the growth and development of *H. contortus* worms and then shorten the survival time of *H. contortus* worms. In the previous discussion, we speculated that a compensatory mechanism may be involved in the regulation of proteasome activity in the egg development experiment and RNAi assay, that is, the downregulation of chymotrypsin activity could promote the activity of other subunits and then enhance the overall activity level of proteasome. It is reported that the proteasome enzyme activity level increases as a whole when *C. elegans* is exposed to oxidative stimulation, which enables the proteasome to degrade aging-related substrate proteins more efficiently and improves the antioxidant ability of *C. elegans*, thereby delaying the worms' aging [1, 74, 75]. Therefore, the decrease of chymotrypsin activity slowed the development of *H. contortus* at the initial stage, but then the worms resumed normal development when the compensatory mechanism and/or negative feedback regulation mechanism participated in the regulation of proteasome activity later if the environment had improved, which prolonged the whole life span of the worms to some extent. However, the specific effects of the *Hc-pbs-5* or chymotrypsin on the life span of *H. contortus* need to be further investigated.

## Conclusion

In this study, the gene encoding proteasomal β5 subunit, named *Hc-pbs-5*, was characterized from the parasitic nematode, and the predicted protein (*Hc*-PBS-5) had core conservative sequences that belong to the N-terminal nucleophile (Ntn) family of hydrolases. *Hc-pbs-5* was transcribed in all key developmental stages with higher levels at L3s as well as adult males of *H. contortus*, and the protein *Hc*-PBS-5 was mainly located in intestine, outer cuticle, muscle cells under the outer cuticle, cervical glands and seminal vesicle of male adults and also in the intestine, outer cuticle, cervical glands, uterine wall, eggs in uterus and ovaries of female adults of *H. contortus*. The inhibitor treatment assay revealed that BTZ could reduce proportions of egg hatching and L4s developed from xL3s of *H. contortus*, respectively. In addition, silencing *Hc-pbs-5* could decrease the transcription of *Hc-pbs-5* and result in fewer xL3s developing to L4s in vitro. Taken together, these results indicate that proteasomal β5 subunit plays an important role in the growth, development and life span of *H. contortus*.

## Supplementary Information

**Additional file 1: Table S1.** Sequences of proteasomal β5 subunits used for phylogenetic and alignment analysis. **Table S2.** Oligonucleotide primers (5’-3’) used in the present study.

## Data Availability

The data supporting the conclusions of this article are provided within the article.

## Abbreviations

aa: Amino acids
BSA: Bovine serum albumin
BTZ: Bortezomib
dsRNA: Double-stranded RNA
EBSS: Earle’s Balanced Salt Solution
IPTG: Isopropyl b-D-1-thiogalactopyranoside
L3s: Third-stage larvae
L4s: Fourth-stage larvae
PBS: Phosphate-buffered solution
RNAi: RNA interference
ssRNA: Single-stranded RNA
UPP: Ubiquitin-proteasome pathway
xL3s: Exsheathed L3s
