# Supplementary material for: A proteasomal β5 subunit of Haemonchus contortus with a role in the growth, development and life span
Source: Parasit Vectors. 2023 Mar 15;16:100. doi: 10.1186/s13071-023-05676-6 (PMC10015785; doi:10.1186/s13071-023-05676-6)
Supplement: Supplementary file 1 — Additional file 1: Table S1. Sequences of proteasomal β5 subunits used for phylogenetic and alignment analysis. Table S2. Oligonucleotide primers (5’-3’) used in the present study. [file 13071_2023_5676_MOESM1_ESM.docx]

Table S1 Sequences of proteasomal β5 subunits used for phylogenetic and alignment analysis.

| Species | GenBank accession number |
| --- | --- |
| *Brugia malayi* | XP_001902498.1 |
| *Caenorhabditis briggsae* | CAP36972.1 |
| *Caenorhabditis elegans*^1^ | CAB04567.1 |
| *Caenorhabditis remanei* | EFO83800.1 |
| *Danio rerio*^1^ | AAH71478.1 |
| *Dictyocaulus viviparus* | KJH44249.1 |
| *Diploscapter pachys* | PAV66776.1 |
| *Drosophila melanogaster* | AAF58748.1 |
| *Homo sapiens*^1^ | NP_002788.1 |
| *Mus musculus*^1^ | NP_035316.1 |
| *Necator americanus*^1^ | ETN68175.1 |
| *Saccharomyces cerevisiae*^1^ | AAA34906.1 |
| *Saccharomyces cerevisiae*^2^ | NP_012533.1 |
| *Toxocara canis* | KHN76925.1 |
| *Xenopus laevis* | XP_018114995.1 |

^1^ Sequence was used for alignment analysis.

^2^ Sequence was used as an outgroup for phylogenetic analysis.

Table S2. Oligonucleotide primers (5’-3’) used in the present study.

| **Primer** | **Sequence (5’-3’) ^a^** |
| --- | --- |
| Primers for isolating the full-length cDNA of *Hc-pbs-5* | |
| Hc-pbs-5-cF | ATGTGGGGTTGTGGTTTTGATGAAC |
| Hc-pbs-5-cR | TCAATCATCGCGAGGTTCGTACGCA |
| Primers for real-time PCR | |
| Hc-pbs-5-rtF | ATAAGCTCCTTCGCCTTTCC |
| Hc-pbs-5-rtR | GTTGTACCCTTCCGAAACTG |
| Hc-tub8-9-rtF | TGTTCCATCACCCAAGGTATCC |
| Hc-tub8-9-rtR | TGACAGACACAAGGTGGTTGAGAT |
| Hc-18s-rtF | AATGGTTAAGAGGGACAATTCG |
| Hc-18s-rtR | CTTGGCAAATGCTTTCGC |
| Primers for prokaryotic expression | |
| Hc-pbs-5-eF | ACCGCGAACATTGGAGGTATGTGGGGTTGTGGTTTTGATGAAC |
| Hc-pbs-5-eR | TCGAATTCGGATCCTCTAGTTCAATCATCGCGAGGTTCGTACGCA |
| Primers for RNA interference | |
| Hc-pbs-5-sF1 | **TAATACGACTCACTATAGGGAGA**CCGGCTACCGCTAACGAACAA |
| Hc-pbs-5-sR1 | AAGCTTCGCAATATCACGACCAAGTT |
| Hc-pbs-5-sF2 | AAGCTTCCGGCTACCGCTAACGAACAA |
| Hc-pbs-5-sR2 | **TAATACGACTCACTATAGGGAGA**CGCAATATCACGACCAAGTT |
| Bt-cry1Ac-sF1 | **TAATACGACTCACTATAGGG**CCAATACAGTACCAGCTACAG |
| Bt-cry1Ac-sR1 | GGATCCGATTCGGCTCTCCACAC |
| Bt-cry1Ac-sF2 | GGATCCCCAATACAGTACCAGCTACAG |
| Bt-cry1Ac-sR2 | **TAATACGACTCACTATAGGG**GATTCGGCTCTCCACAC |
|  |  |

^a^ Underscore represents restriction sites and boldface represents a T7 promoter site.

Hc, *Haemonchus contortus*; Bt, *Bacillus thuringiensis*.
